# The impact of the COVID-19 pandemic on the rate of primary care visits for substance use among patients in Ontario, Canada

**DOI:** 10.1371/journal.pone.0288503

**Published:** 2023-12-21

**Authors:** Colin Siu, Ellen Stephenson, Chelsea D. Christie, Peter Selby, Karen Tu

**Affiliations:** 1 Temerty Faculty of Medicine, Department of Family and Community Medicine, University of Toronto, Toronto, Ontario, Canada; 2 Harvard T.H. Chan School of Public Health, Harvard University, Boston, Massachusetts, United States of America; 3 Campbell Family Research Institute and Krembil Centre for Neuroinformatics, Toronto, Ontario, Canada; 4 Centre for Addiction and Mental Health, Toronto, Ontario, Canada; 5 North York General Hospital, Toronto, Ontario, Canada; 6 Toronto Western Family Health Team, University Health Network, Toronto, Ontario, Canada; Stanford University School of Medicine, UNITED STATES

## Abstract

The COVID-19 pandemic has led to an increase in the prevalence of substance use presentations. This study aims to assess the impact of the COVID-19 pandemic on the rate of primary care visits for substance use including tobacco, alcohol, and other drug use among primary care patients in Ontario, Canada. Diagnostic and service fee code data were collected from a longitudinal cohort of family medicine patients during pre-pandemic (March 14, 2019-March 13, 2020) and pandemic periods (March 14, 2020-March 13, 2021). Generalized linear models were used to compare the rate of substance-use related visits pre-pandemic and during the pandemic. The effects of demographic characteristics including age, sex, and income quintile were also assessed. Relative to the pre-pandemic period, patients were less likely to have a primary care visit during the pandemic for tobacco-use related reasons (OR = 0.288, 95% CI [0.270–0.308]), and for alcohol-use related reasons (OR = 0.851, 95% CI [0.780–0.929]). In contrast, patients were more likely to have a primary care visit for other drug-use related reasons (OR = 1.150, 95% CI [1.080–1.225]). In the face of a known increase in substance use during the COVID-19 pandemic, a decrease in substance use-related primary care visits likely represents an unmet need for this patient population. This study highlights the importance of continued research in the field of substance use, especially in periods of heightened vulnerability such as during the COVID-19 pandemic.

## Introduction

The COVID-19 pandemic has led to increased stress and anxiety as well as maladaptive coping strategies including substance use [1]. One survey indicated that 13% of respondents either initiated or increased substance use to help cope with COVID-19 pandemic stressors [2]. A study showed that 33% of participants increased smoking use [3], while 14% increased alcohol use during the pandemic [4]. Moreover, there have been increases in drug and opioid overdose presentations in hospital emergency departments [5–7].

Primary care providers are ideally positioned to help patients with substance use disorders given their pre-existing relationships with patients. The integration of substance use diagnosis and treatment into primary care is effective in reducing rates of substance use disorders [8,9] and lowering healthcare system costs [10]. However, the COVID-19 pandemic may have led to challenges in administering this type of care. For example, the number of patients attending primary care visits decreased by 34.5% during the pandemic [11]. In particular, preventive care visits were substantially reduced with 89% fewer periodic health exams being conducted [12]. Another challenge is the introduction of telemedicine which represented up to 77.5% of primary care visits during the pandemic [12], and presented unique challenges to substance use care delivery [13,14]. These factors may have served as obstacles to the delivery of high-quality primary care to those with substance use disorders during the COVID-19 pandemic.

The aim of this study was to assess the impact of the COVID-19 pandemic on the rate of primary care visits for substance use including tobacco, alcohol, and other drug use among patients in Ontario, Canada.

## Methods

### Study design and setting

We conducted a longitudinal cohort study of family medicine patients in the University of Toronto Practice-Based Research Network (UTOPIAN) from March 14, 2019, to March 13, 2021. UTOPIAN maintains a primary care electronic medical record (EMR) database with records contributed from over four hundred family physicians in Ontario, Canada. Family physicians contributing data to the UTOPIAN database are more likely to be female, younger, Canadian medical school graduates and practicing in an academic clinic [15]. The majority of patients enrolled in the UTOPIAN database reside in the Greater Toronto Area of Ontario.

Family physician data were included if they had at least 200 rostered patients and their data met UTOPIAN data quality assessment criteria [15]. Patient data were included if they were registered under a family physician that met the above inclusion criteria, had sex and a valid date of birth recorded, had an EMR start date before the study period, and had more than 1 visit in the preceding three years. Patients under 12 years of age were excluded.

The first case of COVID-19 in Ontario was identified on January 25, 2020 [16]. As COVID cases continued to rise, the Ontario government announced a provincial emergency lockdown order on March 17, 2020, which restricted the opening of certain establishments and recreational spaces, limited the size of public gatherings, and closed non-essential businesses [17]. Concurrently, the government of Ontario implemented new service fee codes on March 14, 2020, which remunerated family physicians for the provision of assessments of or counselling to patients by telephone or video [18]. The date of March 14, 2020 was used to define the onset of the COVID-19 pandemic in this study. The University of Toronto Research Ethics Board approved this study (Protocol # 28696/40129).

### Measures

We used two types of measures to identify patients presenting to family physicians with a substance use-related issue: (1) Ontario Health Insurance Plan (OHIP) service fee codes and (2) OHIP diagnostic codes for primary care visits related to substance use. Family physicians are required to submit both an OHIP service fee code for remuneration and an OHIP diagnostic code indicating the primary reason for each patient visit. Substance use visits can be indicated by a substance-use specific service fee code, or a service fee code indicating a general visit alongside a diagnostic code for substance use. Hence, it is important to evaluate both service fee and diagnostic codes when looking for trends in primary care visits.

Ontario service fee codes for tobacco use include: E079 (initial discussion with patient regarding smoking cessation), K039 (smoking cessation follow-up visit), and Q042A (smoking cessation counselling fee) [19]. OHIP diagnostic codes for tobacco use include: 305 (tobacco use), and for alcohol use include: 303 (alcohol use), and 291 (alcoholic psychosis, delirium tremens, Korsakov’s psychosis). OHIP diagnostic codes for other drug use excluding tobacco and alcohol include: 304 (drug dependence, drug addiction), 977 (drug overdose; attempted suicide (drug); adverse effect of drugs and medications–including allergy, overdose, reactions; allergy (drugs and medications)), and 292 (drug psychosis). Both fee codes and diagnostic codes have been used in previous studies to delineate primary care use [20–22].

For both fee and diagnostic code measures, a patient was designated as having had a substance-use-related visit during the period if they had one or more visits that were submitted using the designated service fee or diagnostic codes. Patients with a visit where both a substance use-related fee code and a diagnostic code were submitted were only counted once. To estimate the rate of patients with at least one substance-use related visit within each time period, the total number of patients with a substance-use-related visit was divided by the number of eligible patients within that period.

### Statistical analysis

We used a series of generalized linear models to compare the rate of substance-use related fee and diagnostic codes pre-pandemic (March 14-2019-March 13, 2020) and during the pandemic (March 14, 2020-March 13, 2021). All models assessing the impact of the pandemic on tobacco, alcohol, and other drug use related visits were adjusted for age, sex, and neighborhood income quintile. Logistic regression was used to model the binary outcome of whether a substance-use related visit took place in either period. For the outcome measures, we assessed the effects of demographic characteristics including age, sex, neighborhood income quintile (based on postal code) [23], and pre-pandemic/pandemic periods, as well as possible interactions between these characteristics. Generalized estimating equations were used to estimate the model parameters (GEE). We used an exchangeable correlation structure to account for within-patient correlation over the two periods. We calculated two-sided p-values and 95% confidence intervals for the changes in rate of primary care visits during the pre-pandemic and pandemic periods. Statistical significance was based on two-sided p-values at the 0.05 level. Analyses were performed in R version 4.1.1.

## Results

234,730 patients and 240,044 patients met eligibility criteria for study inclusion in the pre-pandemic and pandemic periods, respectively. Of these, 217,913 patients were eligible and contributed data to both pre-pandemic and pandemic periods. Patients between the ages of 50–64 years made up the largest age category in both periods. The study population composed of slightly more female than male patients. Consistent with past research using UTOPIAN EMR data, patients in the highest income quintile made up the largest percentage of patients [15]. The distributions across age groups, income quintiles, and sex were similar in pre-pandemic and pandemic periods (Table 1).

**Table 1:** Demographics of eligible study patients in the pre-pandemic and pandemic periods.

	Pre-Pandemic Period (14 March 2019 to 13 March 2020) n = 234,730 Patients	Pandemic Period (14 March 2020–13 March 2021) n = 240,044 Patients
Age		
12–18 years	7.6% (17,729)	8.4% (20,282)
19–34 years	21.4% (50,211)	21.5% (51,654)
35–49 years	23.6% (55,406)	23.3% (55,944)
50–64 years	25.4% (59,536)	25.1% (60,243)
65 years and older	22.1% (51,848)	21.6% (51,921)
Neighborhood income quintile		
Lowest quintile	21.0% (49,401)	21.1% (50,761)
Middle-low quintile	17.0% (39,843)	17.1% (40,986)
Middle quintile	15.8% (37,130)	15.9% (38,104)
Middle-high quintile	17.7% (41,481)	17.7% (42,455)
Highest quintile	25.1% (58,992)	25.0% (59,918)
Missing data	3.4% (7,883)	3.3% (7,820)
Sex		
Male	43.1% (101,070)	42.8% (102,674)
Female	56.9% (133,660)	57.2% (137,370)

**Fig 1:**
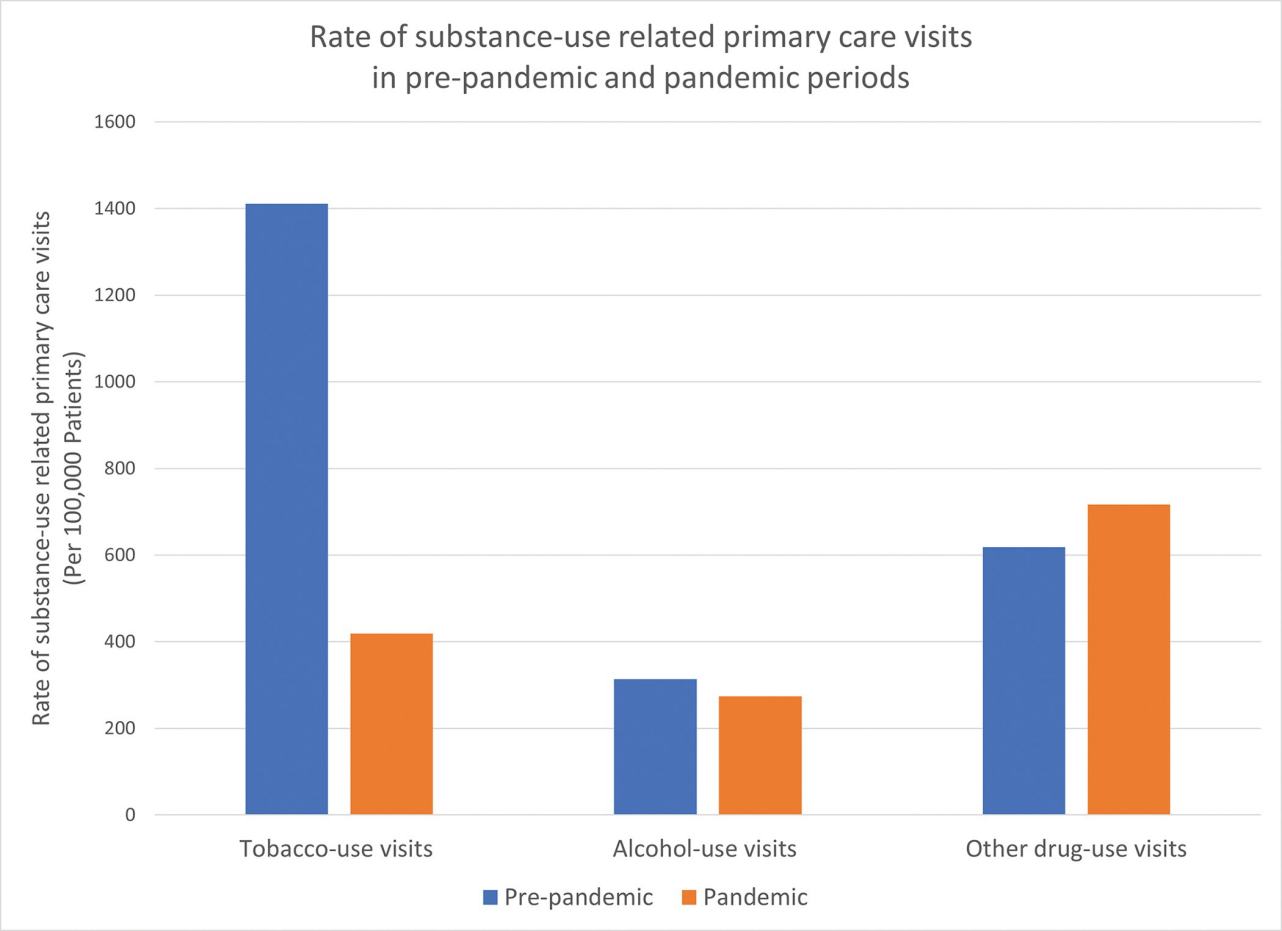
Rate of substance-use related primary care visits in pre-pandemic and pandemic periods.

Across all measures, except visits for other drug use, the rate of primary care visits for substance use-related reasons decreased overall from the pre-pandemic to the pandemic period (Table 2; Fig 1). Relative to the pre-pandemic period, there were decreases during the pandemic in the proportion of patients who had a primary care visit for tobacco-use related reasons from 1411 to 419 per 100,000 patients and alcohol-use related reasons from 314 to 274 per 100,000 patients. However, the proportion of patients having a primary care visit during the pandemic compared to the pre-pandemic period for other drug-use related reasons increased from 618 to 717 per 100,000 patients.

**Table 2:** Rate of substance-use related primary care visits in pre-pandemic and pandemic periods.

Measure	Rate pre-pandemic per 100,000 patients*	Rate during pandemic per 100,000 patients*	Change in rate from pre-pandemic to pandemic periods	p-value
Tobacco-use visits based on service fee or diagnostic codes	1,411 (3312)	419 (1005)	(-) 70.33%	< .0001
Alcohol-use visits based on diagnostic codes	314 (738)	274 (658)	(-) 12.81%	0.0003
Other drug-use visits based on diagnostic codes	618 (1451)	717 (1720)	(+) 15.91%	< .0001

*Measured as rate of patients with one or more visits with the respective fee/diagnostic code in that period per 100,000 patients (visit count in parentheses). The number of eligible patients present in the pre-pandemic period was 234,730 and in the pandemic period was 240,044.

After adjustment for age, sex, and neighbourhood income, the pandemic period was associated with lower odds of having a tobacco use-related visit (OR = 0.288, 95% CI: 0.270–0.308) or an alcohol use-related visit (OR = 0.851, 95% CI: 0.780–0.929), but with higher odds of having a visit related to other drug use (OR = 1.150, 95% CI: 1.080–1.225).

With regards to tobacco use, patients who were aged 12–18 years were least likely to have a primary care visit, whereas patients aged 50–64 years were most likely to have a visit. Alcohol-use related visits were most common among patients aged 50–64 years. Males were more likely than females to have all types of substance-use related visits, while patients in the highest income quintile were the least likely to have other drug-use related visits. During the pandemic, sex became a slightly weaker predictor of the risk of other drug-use related visits than it was before the pandemic. There were no significant changes in age-related or income quintile differences in substance-use visits between pre-pandemic and pandemic periods (S1 Table, S1–S3 Figs).

## Discussion

Although substance use increased during the pandemic [2], the rate of primary care visits for most substance use-related presentations decreased from the pre-pandemic to the pandemic period. Our study found that tobacco-use and alcohol-use related visits decreased during the pandemic by 71% and 13%, while other drug-use related visits increased by 16%. In an earlier study we found that in the first nine and a half months of the pandemic, periodic health exams decreased by 89% while visits for common chronic diseases such as diabetes and hypertension decreased by 25% [12]. Alcohol-use related visits may have decreased as patients may have perceived their alcohol use as being within societal expectations in the context of the pandemic and hence, they may have not actively sought care. Lastly, periodic health exams are often an opportunity to review a patient’s smoking status and alcohol intake: issues which may not otherwise arise during visits for other acute concerns. Hence, the decrease in periodic health exams in the pandemic [11] may have contributed to the decline in smoking and alcohol use visits reported in our study. Contrastingly, other drug-use related visits increased during the pandemic. These other drug-use related visits include treatment of opioid use and stimulant use disorders. The increase in other drug-use related visits noted during the pandemic may reflect the increased opioid use prevalence noted during the pandemic and the requirement for continued primary care visits for prescribing of opioid agonist therapy [24,25].

One of the primary drivers of reduced visits may be the hesitancy of patients to seek health care during the pandemic due to fears of acquiring a COVID-19 infection [26]. Furthermore, at the beginning of the pandemic, there was a shortage of personal protective equipment to allow providers to see patients and many providers were redeployed to COVID-19 care [6]. Altogether, these factors may have contributed to decreases in the number of in-person visits [12,27,28], and increases in the number of telemedicine visits during the pandemic [28]. It is important to note that patients with substance use disorders are more likely to face structural health barriers which results in inequities regarding accessibility to telemedicine services [29–31]. In addition, telemedicine may not be optimal for the delivery of preventive care such as smoking cessation. For example, one study indicated that preventive diagnoses made up 25.6% of in-person based care but only 2.7% of telemedicine care during the pandemic [32].

The decrease in rate of primary care visits for alcohol-use and tobacco-use presentations can be juxtaposed with an increase in substance use during the pandemic. An increase in alcohol use and alcohol withdrawal hospital admissions were noted during the pandemic [4,33,34], and a similar increase was seen with smoking and other drug use [35–38]. Increased substance use may be used as a coping mechanism for stressors associated with the pandemic [2,37]. The contrast between an increase in substance use prevalence and a decrease in substance-use related primary care visits during the pandemic likely represents an unmet need for this patient population.

The results of our analysis of associations between demographic characteristics and substance-use related visits are consistent with the literature around substance use prevalence. Younger patients were least likely to have substance-use related primary care visits while those that were middle-aged were most likely to have such visits [39–41]. Those in the lowest income quintile were most likely to have an other drug-use related visit while those in the highest income quintile were least likely to do so [42–44]. Lastly, males were more likely than females to have substance-use related primary care visits [39,40,45–51].

### Limitations of the study

Our study is limited by the use of service fee codes and diagnostic codes as surrogate measures for primary care use. Furthermore, our study did not evaluate for repeat visits by patients during the study period. Future research may want to consider whether patients with existing substance use issues had increases or decreases in the rate of substance use visits during the pandemic. In addition, the new telemedicine fee codes implemented on March 14, 2020 could be used for remuneration of telemedicine visits related to any family medicine presentation including substance use. Hence, family physicians may have used have these telemedicine fee codes in lieu of substance-use specific service fee codes. However, the use of diagnostic codes as a complimentary measure of primary care use reduces the risk of misclassification bias because both telemedicine and in-person visits require the use of the same OHIP diagnostic codes. Nevertheless, if providers discuss multiple issues including substance use during a single visit, they have a choice in which diagnostic codes to report. Thus, both diagnostic and fee codes are reliant on providers’ self-report. Finally, our study examines the effects of the COVID-19 pandemic up to March 14, 2021 during a period of increased social and medical vulnerability. Future studies may consider examining the long-term trends of substance-use related primary care visits beyond this period.

## Conclusion

Our study uses service fee codes and diagnostic codes obtained from primary care visits to evaluate the rate of substance-use related visits in pre-pandemic and pandemic periods. Our results show that substance-use related primary care visits decreased in the first year of the COVID-19 pandemic relative to the year before. Our results, interpreted in an environmental context of increased substance use during the COVID-19 pandemic, likely represent an unmet need for patients with substance use disorders during the pandemic. This study contributes to a growing body of literature on the impact of the COVID-19 pandemic on substance-use related health care and primary care health systems.

## Supporting information

S1 Table Comparison of substance-use related primary care visits in pre-pandemic periods based on age, neighborhood income quintile, and sex. (DOCX)

S1 Fig Rate of substance-use related primary care visits in pre-pandemic and pandemic periods by age categories. (DOCX)

S2 Fig Rate of substance-use related primary care visits in pre-pandemic and pandemic periods by neighborhood income quintiles. (DOCX)

S3 Fig Rate of substance-use related primary care visits in pre-pandemic and pandemic periods by sex. (DOCX)
