# Supplementary material for: The impact of the COVID-19 pandemic on the rate of primary care visits for substance use among patients in Ontario, Canada
Source: PLoS One. 2023 Dec 21;18(12):e0288503. doi: 10.1371/journal.pone.0288503 (PMC10734921; doi:10.1371/journal.pone.0288503)
Supplement: S1 Table — (DOCX) [file pone.0288503.s001.docx]

**S1 Table: Comparison of substance-use related primary care visits in pre-pandemic periods based on age, neighborhood income quintile, and sex.**

|  | Measure | Rate Pre-pandemic per 100,000 patients* | Rate during pandemic per 100,000 patients* | Change in rate from pre-pandemic to pandemic periods |
| --- | --- | --- | --- | --- |
| 12-18 years | Tobacco-use visits  Alcohol-use visits  Other drug-use visits | 15.8 (37)  0.9 (2)  24.7 (58) | 3.7 (9)  2.1 (5)  25.4 (61) | (-) 76.21%  (+) 144.47%  (+) 2.84% |
| 19-34 years | Tobacco-use visits  Alcohol-use visits  Other drug-use visits | 208.3 (489)  55.0 (129)  122.7 (288) | 62.5 (150)  51.7 (124)  142.5 (342) | (-) 70.00%  (-) 6.00%  (+) 16.12% |
| 35-49 years | Tobacco-use visits  Alcohol-use visits  Other drug-use visits | 375.3 (881)  89.5 (210)  154.6 (363) | 121.2 (291)  79.6 (191)  164.1 (394) | (-) 67.70%  (-) 11.06%  (+) 6.14% |
| 50-64 years | Tobacco-use visits  Alcohol-use visits  Other drug-use visits | 577.7 (1356)  116.3 (273)  167.0 (392) | 156.2 (375)  93.7 (225)  193.3 (464) | (-) 72.96%  (-) 19.41%  (+) 15.75% |
| 65 years and older | Tobacco-use visits  Alcohol-use visits  Other drug-use visits | 233.9 (549)  52.8 (124)  149.1 (350) | 75.0 (180)  47.1 (113)  191.2 (459) | (-) 67.94%  (-) 10.89%  (+) 28.24% |
| Lowest quintile | Tobacco-use visits  Alcohol-use visits  Other drug-use visits | 428.6 (1006)  81.8 (192)  173.0 (406) | 120.0 (288)  59.6 (143)  188.7 (453) | (-) 72.01%  (-) 27.17%  (+) 9.11% |
| Middle-low quintile | Tobacco-use visits  Alcohol-use visits  Other drug-use visits | 279 .5 (656)  53.3 (125)  103.9 (244) | 94.1 (226)  49.6 (119)  138.7 (333) | (-) 66.3%  (-) 6.91%  (+) 33.45% |
| Middle quintile | Tobacco-use visits  Alcohol-use visits  Other drug-use visits | 222.4 (522)  46.9 (110)  90.3 (212) | 70.8 (170)  40.0 (96)  98.3 (236) | (-) 68.15%  (-) 14.66%  (+) 8.86% |
| Middle-high quintile | Tobacco-use visits  Alcohol-use visits  Other drug-use visits | 186.6 (438)  52.8 (124)  99.7 (234) | 52.9 (127)  48.7 (117)  109.6 (263) | (-) 71.65%  (-) 7.73%  (+) 9.91% |
| Highest quintile | Tobacco-use visits  Alcohol-use visits  Other drug-use visits | 256.5 (602)  70.7 (166)  117.2 (275) | 67.9 (163)  66.2 (159)  146.2 (351) | (-) 73.52%  (-) 6.34%  (+) 24.81% |
| Female | Tobacco-use visits  Alcohol-use visits  Other drug-use visits | 600.7 (1410)  110.8 (260)  295.2 (693) | 167.9 (403)  105.8 (254)  387.0 (929) | (-) 72.05%  (-) 4.47%  (+) 31.09% |
| Male | Tobacco-use visits  Alcohol-use visits  Other drug-use visits | 810.3 (1902)  203.6 (478)  322.9 (758) | 250.8 (602)  168.3 (404)  329.5 (791) | (-) 69.05%  (-) 17.35%  (+) 2.04% |

*Measured as rate of patients with one or more visits with the documented respective fee/diagnostic code in that period per 100,000 patients (visit count in parentheses). The number of eligible patients present in the pre-pandemic period was 234,730 and in the pandemic period was 240,044.
