# Supplementary material for: The impact of the COVID-19 pandemic on the rate of primary care visits for substance use among patients in Ontario, Canada
Source: PLoS One. 2023 Dec 21;18(12):e0288503. doi: 10.1371/journal.pone.0288503 (PMC10734921; doi:10.1371/journal.pone.0288503)
Supplement: S3 Fig — (DOCX) [file pone.0288503.s004.docx]

**S3 Figure: Rate of substance-use related primary care visits in pre-pandemic and pandemic periods by sex.**
